# Unusual Motor Hand Neuropathies: Causes, Diagnosis, and Evaluation of Motor Impairments

**DOI:** 10.7759/cureus.66381

**Published:** 2024-08-07

**Authors:** Vasily Khodulev, Artsiom Klimko, Olga Pereverzeva, Nataliya Charnenka, Tatsiana Hryharovich, Nadezhda Kabirova, Hanna Khoduleva

**Affiliations:** 1 The Functional Diagnostics Department, Republican Research and Clinical Center of Neurology and Neurosurgery, Minsk, BLR; 2 Department of Neurology, University Hospital of Zurich, Zurich, CHE; 3 Consultative-Polyclinic Department, Republican Research and Clinical Center of Neurology and Neurosurgery, Minsk, BLR; 4 Ultrasound Diagnostics, Multidisciplinary Medical Center “Healthy Sleep Center”, Minsk, BLR; 5 Ultrasound Diagnostics, Mayak Zdorovia, Minsk, BLR; 6 Department of Neurology, Gomel Regional Children’s Clinical Hospital, Gomel, BLR

**Keywords:** recurrent motor branch of the median nerve, deep motor branch of the ulnar nerve, hand neuropathies, ultrasound imaging, nerve conduction studies, isolated injury

## Abstract

Background

Isolated hand motor nerve injuries, specifically those affecting the recurrent motor branch of the median nerve and the deep motor branch of the ulnar nerve, are rarely reported in medical literature. Diagnosing and quantifying these injuries pose significant challenges due to their uncommon nature and the variety of mechanisms that can cause them.

Methodology

This study reviews six unusual cases of isolated damage to the recurrent motor branch of the median nerve and the deep motor branch of the ulnar nerve, including cases with combined injuries. The etiologies include various traumatic and compressive mechanisms, such as a blow from the thenar to the back of a knife blade, long-distance cycling, impact from a broken shovel handle, knife injury, and damage from a screw while using a cordless screwdriver. In one case, the cause was indeterminate. Diagnostic methods involved clinical evaluation, electrophysiological testing (nerve conduction studies and electromyography), and high-resolution ultrasound imaging. A thorough medical history was also crucial in understanding the injury mechanisms.

Results

The cases demonstrated a range of causes for isolated hand motor nerve injuries, with both traumatic and compressive mechanisms identified. The diagnostic process highlighted the value of integrating clinical assessments, electrophysiological data, and ultrasound imaging to accurately diagnose and understand the extent and nature of the injuries.

Conclusions

Isolated motor nerve injuries in the hand can arise from diverse and often unexpected causes. Comprehensive clinical evaluation, supported by electrophysiological testing and ultrasound imaging, is essential for accurate diagnosis and management. A detailed medical history is invaluable in identifying the mechanism of injury, which is critical for developing an appropriate treatment plan. The study underscores the importance of a multidisciplinary approach in diagnosing and treating these rare neuropathies.

## Introduction

Carpal tunnel syndrome (CTS), characterized by damage to the median nerve, is the most prevalent neuropathy affecting the wrist and hand. Neuropathy of the ulnar nerve in Guyon’s canal is less frequently observed compared to CTS. While CTS and ulnar tunnel syndrome are well-known conditions, other less prevalent neuropathies can affect the hand by disrupting the distal branches of the motor fibers of the median and ulnar nerves [1-4]. Specifically, isolated damage of the recurrent motor branch (RMB) of the median nerve or the deep motor branch of the ulnar nerve (DBUN) is seldom documented in medical literature, and there is a significant delay in diagnosis.

Isolated RMB and DBUN neuropathies, though rare, have substantial clinical significance due to their impact on hand function. The RMB of the median nerve primarily innervates the thenar muscles, which are crucial for thumb opposition, abduction, and flexion. Damage to the RMB can impair grip strength and dexterity, hindering activities that require fine motor skills. Similarly, the DBUN innervates most of the intrinsic hand muscles, including those responsible for finger abduction and adduction, as well as some of the muscles involved in the flexion of the fourth and fifth fingers. Neuropathy of the DBUN can result in weakened hand grip, reduced finger coordination, and difficulty performing tasks that require precise hand movements.

For a precise diagnosis of the level of peripheral nerve damage, it is imperative to employ clinical data, conduct a thorough anamnesis, and complement these efforts with appropriate diagnostic techniques [5,6]. The combination of nerve conduction studies (NCSs) and ultrasound imaging (USI) is recognized as the gold standard for diagnosing peripheral neuropathies. In this study, we present rare instances of isolated damages to the RMB of the median nerve and the DBUN, as well as combinatorial injuries of these nerves. This study aims to investigate the etiological factors, mechanisms, and diagnostic challenges of such injuries. We seek to highlight the importance of early and accurate diagnosis, as well as tailored treatment approaches, to improve patient outcomes in these complex neuropathies.

## Materials and methods

This study was conducted at the Functional Diagnostics Department of the Republican Research and Clinical Center of Neurology and Neurosurgery (Minsk, Belarus). We retrospectively reviewed patients who were treated at our center from January 2021 until June 2023, identifying a total of six patients with suspected isolated motor nerve injuries of the hand. Inclusion criteria were patients aged 18-65 years with clinical signs and symptoms suggestive of isolated damage to the RMB of the median nerve or the DBUN. Exclusion criteria included patients with a history of systemic neuropathies, previous surgeries on the affected hand, or other conditions that could confound the diagnosis.

Data collection involved a thorough medical history taken by standardized questionnaires administered by the first author (VK). This included details about the onset and progression of symptoms, occupational and leisure activities, and any relevant incidents before the onset of symptoms. Participants were recruited retrospectively from the medical records of patients treated at the Functional Diagnostics Department between January 2021 and June 2023. Patients who met the inclusion criteria and had sufficient medical documentation were included in the study. Physical examination via local inspection looked for signs of muscle atrophy and/or the presence of tenderness upon palpation in the hand, carpal tunnel, or Guyon’s canal. Motor testing evaluated intrinsic and extrinsic muscles in both median and ulnar distributions. Muscle strength was determined using the Medical Research Council (MRC) scale. The presence or absence of hand sensitivity was also assessed. Special tests included Tinel’s, Durkan’s, Phalen’s, reverse Phalen’s, Wartenberg’s, Froment’s, and Egawa’s signs. All physical examinations were performed by the first author (VK).

Neurophysiological studies were conducted using the VikingSelect neurodiagnostic system (ViasysHealthcare, Nicolet Biomedical). Standard techniques with surface recording Ag/AgCl disk electrodes (10 mm diameter) were used for motor NCSs. Amplifier filters were set between 2 Hz and 10 kHz. The median compound muscle action potential (CMAP) was recorded from the abductor pollicis brevis (APB) with active electrodes placed over the motor point and reference electrodes positioned distally over the proximal phalanx of the first digit (a belly-tendon montage). The median nerve was stimulated at 2 cm proximal to the distal wrist crease and the antecubital fossa. In addition, two-channel median motor NCSs of the APB and the second lumbrical (SL) muscles were performed. The active recording electrode over the SL muscle was placed slightly lateral to the midpoint of the third metacarpal bone, with the reference electrode over the proximal phalanx of the second digit. This two-channel technique assessed the segments of the median motor nerve to the APB and SL. The ulnar CMAPs were recorded from the ulnar-innervated abductor digiti minimi (ADM), deeper interossei muscles, and the first dorsal interosseous (FDI), with active electrodes placed over motor points of respective muscles. The active recording electrode over deeper interossei muscles was positioned as for the SL. The ulnar nerve was stimulated at 2 cm proximal to the distal wrist crease and below and above the elbow. Sensory nerve action potentials (SNAPs) were obtained antidromically by surface stimulation of median and ulnar nerves at the proximal wrist crease and recording from the thumb, index, middle (median nerve), ring (median and ulnar), and fifth (ulnar) fingers with ring electrodes. Amplifier filters were set between 20 Hz and 3 kHz. Distal motor latency, conduction velocity, and peak-to-peak amplitude of CMAP and SNAP were analyzed.

USI was performed using ultrasound devices Vivid E 9 (GE), HD 11 XE (Philips), and EPIQ 7 (Philips), utilizing linear transducers operating within a frequency range of 5-12 MHz and 4-18 MHz. Patients were examined in both supine and seated positions. The study extended beyond mere visualization of the nerve of interest, encompassing the spinal nerves and the nerves in the supraclavicular and infraclavicular portions of the brachial plexus. This comprehensive approach was necessary to exclude multi-level nerve damage. Additionally, muscles corresponding to the innervation zone and blood vessels along the nerve paths were evaluated. USI of analogous anatomical structures on the contralateral side was performed in all cases. For visualizing nerve trunks and soft tissues, a grayscale (two-dimensional) mode was used, while color Doppler modes were employed for visualizing blood vessels. Nerve trunk scanning was conducted in both longitudinal (long axis) and transverse (short axis) projections. Continuity, contour, structure, echogenicity of the nerve trunk, signs of muscle hypotrophy, and vascular pathology were evaluated. Nerve size and echogenicity were assessed in relation to the contralateral side.

Data from medical history, NCS, and USI were systematically analyzed. For NCS, parameters such as distal motor latency, conduction velocity, and peak-to-peak amplitude of CMAP and SNAP were measured and compared to normative values. USI data included evaluations of nerve continuity, contour, structure, echogenicity, and measurements of nerve trunk and muscle thickness compared to the contralateral side. Statistical analyses, including descriptive statistics, were used to summarize findings and draw conclusions. All analyses were performed using standard statistical software.

This study was conducted in accordance with the Declaration of Helsinki and was approved by the Ethics Review Committee (ERC) of the Republican Research and Clinical Center of Neurology and Neurosurgery (Minsk, Belarus). Informed consent was obtained from all participants involved in the study. Data analysis included both qualitative descriptions of clinical findings and quantitative measures from NCS and USI, with statistical comparisons made to evaluate treatment outcomes.

## Results

Case 1: Traumatic neuropathy of the recurrent motor branch of the median nerve induced by a blow by the thenar to the back of a knife blade

**Figure 1:**
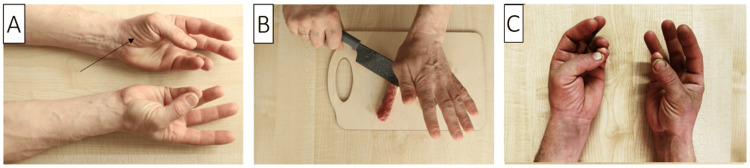
(A) Profound atrophy of the lateral thenar muscles on the left (indicated by an arrow) and the inability to join the thumb and little finger are observed. (B) Mechanism of damage to the motor branch of the median nerve. (C) Partial recovery of the opposition of the thumb.

A 42-year-old, right-handed male, working as an automobile mechanic, reported a seven-month history of reduced dexterity, pinch and grasp function, and limited movement of the left thumb, accompanied by thenar muscle atrophy. There was no pain in the thenar eminence. He denied any previous trauma or infections and did not experience localized hand, neck, or upper limb pain. His treatment included ipidacrine and alpha-lipoic acid, complemented by physical therapy. The neurological assessment revealed significant atrophy of the lateral thenar muscles, an inability to maintain the thumb in a palmar abducted position (thumb abduction power rated at 0/5), and a failure to oppose the thumb to the fifth finger (Figure 1). Sensory function in the left hand and fingers remained intact. The Durkan, Phalen, reverse Phalen, and Tinel tests were negative. The observed atrophy was further confirmed through NCSs and USI.

**Figure 2:**
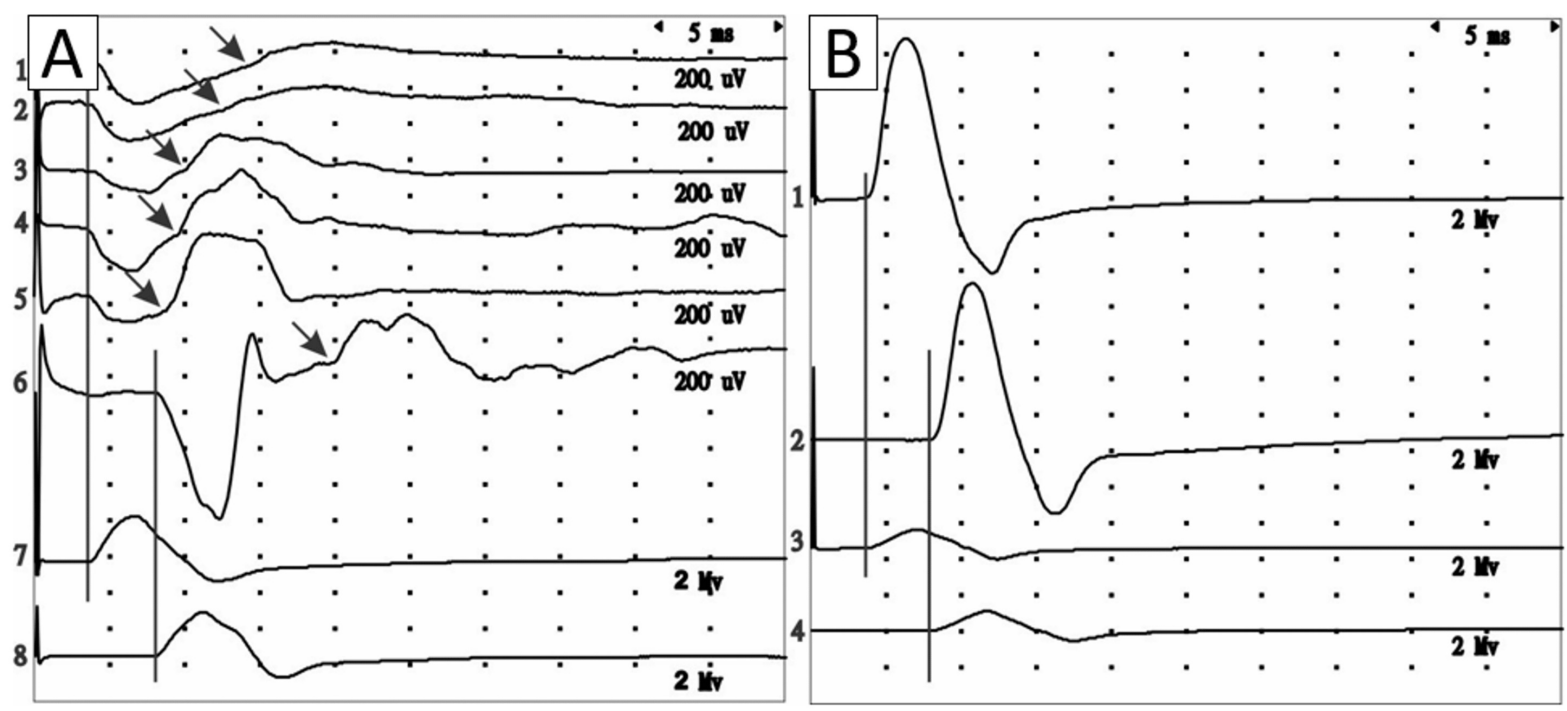
CMAPs recorded from the APB (traces 1A-6A, 1B-2B) and SL (traces 7A-8A, 3B-4B) muscles following distal (1A-5A, 7A) and proximal (6A, 8A, 2B, 4B) stimulation of the left (A) and right (B) median nerves. (A) Traces 1A-6A recorded at 7, 11, 17, 21, and 24 months of the disease, respectively. Vertical lines show the same latency deviation of the curves from the baseline recorded from APB and SL at distal and proximal stimulation. An arrow indicates the onset latency of the CMAPs. CMAP: compound muscle action potential; APB: abductor pollicis brevis; SL: second lumbrical

Motor conduction studies indicated pronounced axonal damage to the left median motor fibers to the APB (Table 1). Median CMAP was absent when recorded over the APB muscle (initial positive signal deviation), but normal when recorded over the SL muscle (Figure 2). Sensory recordings and ulnar NCS were within normal limits. MRI of the cervical spine did not show any signs of cervical myelopathy or cervical root compression.

**Table 1:** Results of serial motor NCSs at different times in relation to symptom onset. NCS: nerve conduction study; DML: distal motor latency; CV: conduction velocity; APB: abductor pollicis brevis; SL: second lumbrical; ND: not done

Muscle, month of disease	DML, ms	Duration, ms	Amplitude, mV	Area, mVms	CV, m/s
Left APB, 7	14.9	7.9	0.1	0.7	ND
Left APB, 11	12.5	11.1	0.1	1.0	ND
Left APB, 17	10.1	8.1	0.3	1.6	ND
Left APB, 21	9.3	7.9	0.3	1.8	19.0
Left APB, 24	8.8	7.6	0.4	2.5	19.0
Left SL	3.6	6.5	3.7	9.1	50.0
Right APB	3.8	5.5	13.0	28.5	51.0
Right SL	3.9	6.7	1.6	3.8	51.0

**Figure 3:**
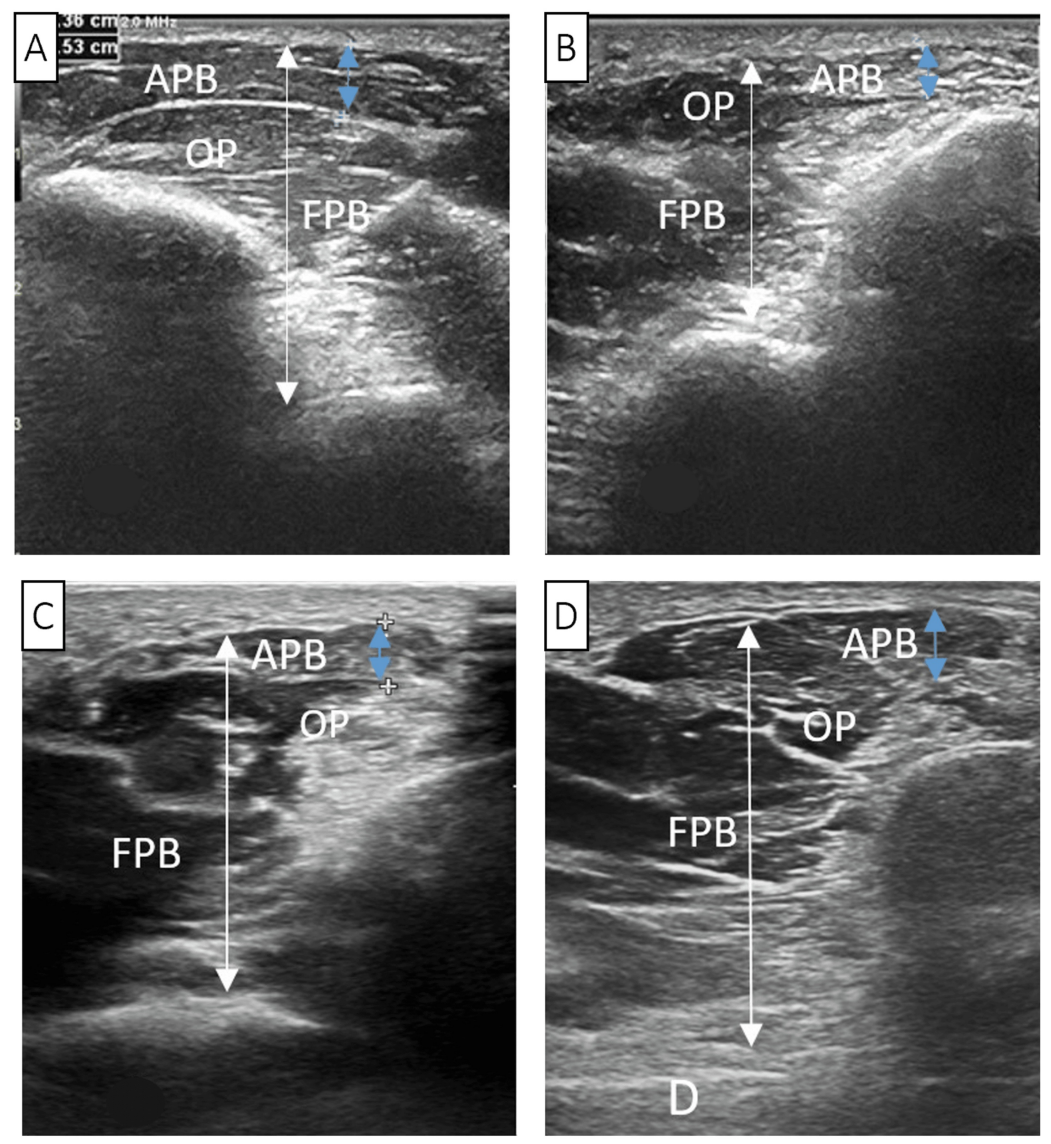
Ultrasound imaging of the muscles of the right (A, normal imaging) and left (B, C, D) thenar eminence of the thumb at 7 (B), 17 (C), and 21 (D) months of the disease. The ultrasound probe is positioned over the thenar region, perpendicular to the first metacarpal bone. The white arrows indicate the total thickness of the thenar muscles, and the blue arrows indicate the thickness of the APB. APB: abductor pollicis brevis; OP: opponens pollicis; FPB: flexor pollicis brevis

USI revealed increased echogenicity in the left APB muscle, with a reduction in thickness to 3.6 mm compared to the right hand’s 5.3 mm, and the total thickness of all thenar muscles was 21 mm and 32 mm, respectively (Figures 3A, 3B). Measurements of the thickness of the opponens pollicis (OP) and the flexor pollicis brevis (FPB) were not performed due to indistinct boundaries between these muscles. These observations were consistent with ultrasonographic indicators of muscle atrophy. No changes were identified in the spinal cord nerves, brachial plexus nerves, median nerves in the shoulder or forearm, or within or at the exit of the carpal tunnel. The RMB was not discernible using a 5-12 MHz frequency probe.

On the left hand, USI revealed significant ultrasonographic characteristic changes in the thenar muscles, predominantly the APB: the muscles had an unclear contour, unevenly increased echogenicity, and disrupted structure. The thickness of the APB was reduced to 3.6 mm compared to 5.3 mm on the right hand, and the total thickness of all thenar muscles was 21 mm and 32 mm, respectively. Measurements of the thickness of the OP and the FPB were not performed due to indistinct boundaries between these muscles. These observations were consistent with ultrasonographic signs of muscle atrophy. No changes were detected in the spinal cord nerves, nerves of the brachial plexus, median nerves in the shoulder and forearm, or within or at the exit of the carpal tunnel. The RMB was not discernible using a probe with a frequency range of 5-12 MHz.

Upon further questioning within a month and repeated emphasis that the potential nerve injury was at the level of the carpal joint or thenar area, the patient recalled that he had been regularly chopping chicken necks for his cat for three months before the onset of symptoms. During this action, he struck with the area of maximum thenar eminence to the back of a knife blade, but sometimes he missed it (Figure 1B). In this case, a strong sharp pain occurred, which went away after a few minutes. Based on the detailed medical history, clinical and electrophysiological data, and USI, a diagnosis of severe isolated traumatic RMB neuropathy of the median nerve, affecting the thenar muscles, was made.

Subsequently, the patient underwent a course of repetitive peripheral magnetic stimulation, focusing on the thenar region and the area somewhat proximal to the thenar along the median nerve. Eleven months after symptom onset and two months post-treatment, a follow-up examination revealed improved hand function, enabling the patient to touch the thumb to the fifth digit. By 17 months post-injury, the patient noted further hand function improvements, and by the 21st month, he reported no significant complaints. He could connect the thumb with the fifth finger, but he still could not do palmar abduction of the thumb away from the plane of the palm (Figure 1C).

Seventeen months from the initial symptoms, NCSs indicated the emergence of CMAP with low amplitude and increased distal latency to the APB muscle. These responses manifested at the end of the positive phase of the curve (Figure 2A). By 24 months, CMAP amplitude increased slightly, and distal latency decreased (Table 1). During proximal stimulation, a positive deflection preceding CMAP was recorded, the latency of which coincided with the latency of CMAP from the SL muscle. A retrospective analysis of earlier recordings revealed that a very low CMAP had been recorded previously at seven and eleven months of illness, respectively (Figure 2). The patient likely experienced improvement due to a combination of repetitive peripheral magnetic stimulation, medications (ipidacrine and alpha-lipoic acid), and physical therapy, which collectively contributed to the gradual recovery of nerve function.

USI in the 17th month also reflected positive dynamics, showing an increase in the APB’s thickness to 4.3 mm and a more differentiated structure, albeit with increased echogenicity compared to the right hand. The delineation between individual muscles became clearer. Parts of the muscle with fibrous changes and others with a normal echo structure were identified in the OP and FPB, with fibrous changes more evident in the APB. The total thickness of the thenar muscles increased from 21 mm to 24 mm. By the 21st month, the APB’s structure and echogenicity remained unchanged, but its thickness increased to 4.5 mm. The thenar’s total thickness reached 28 mm, attributed solely to the OP. The areas of increased echogenicity in the OP and FPB muscle bellies diminished.

**Figure 4:**
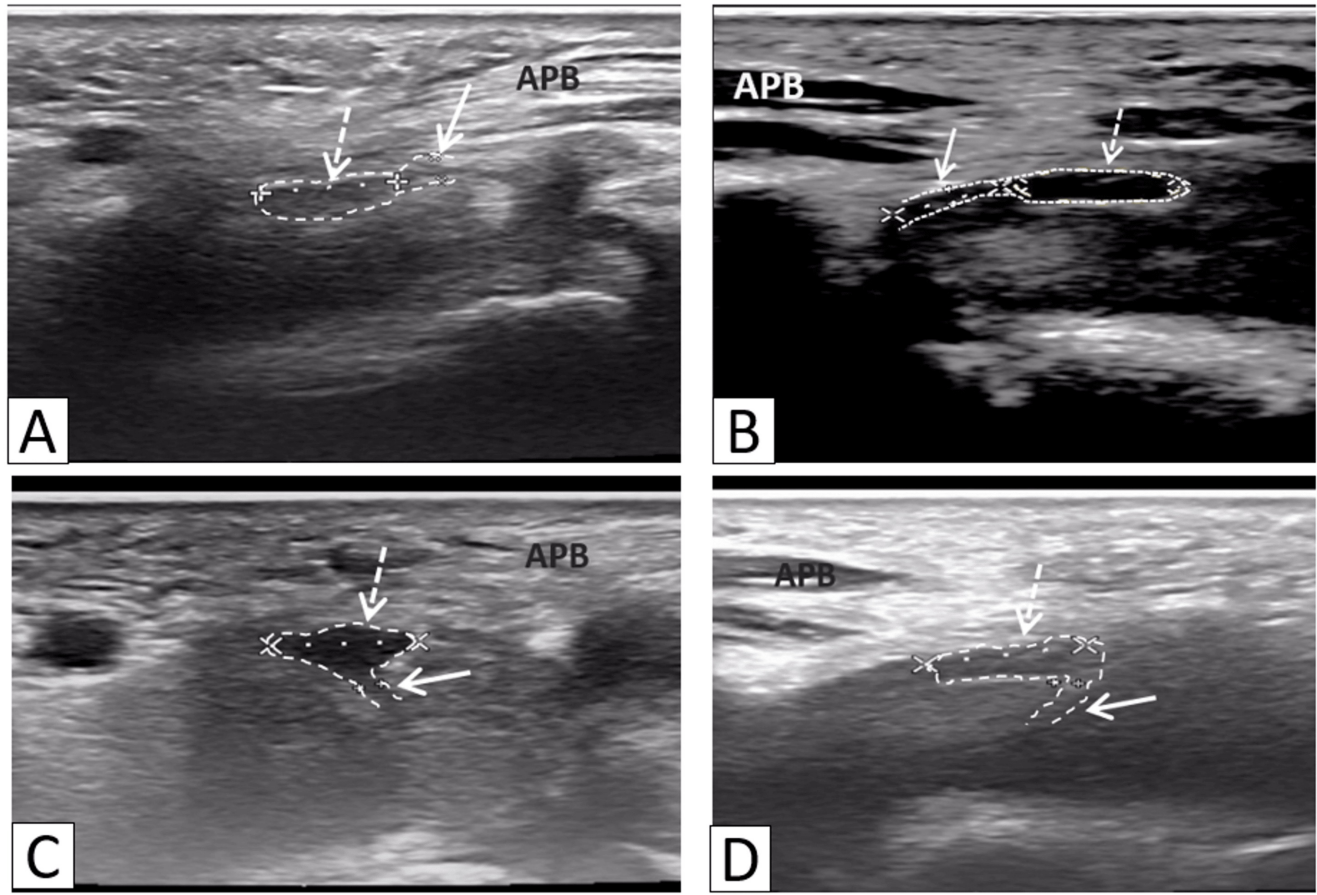
Transverse 18–4-MHz ultrasound imaging of the median nerve (dashed arrow) at the level of the origin of the left (A) and right (B) recurrent motor branch (solid arrow), and at the level of the origin of the left (C) and right (D) branches to the first and second lumbrical muscles (solid arrow). The ultrasound probe is positioned over the thenar region, perpendicular to the first metacarpal bone. This ultrasound image demonstrates an enlarged recurrent motor branch (A, solid arrow) arising from the radial division of the median nerve (dashed arrow).

At the 24th month of the disease, ultrasound with linear transducers operating at frequencies between 4 and 18 MHz revealed a left RMB with a length of 3.3 mm, an increased transverse long-axis diameter of 1.6 mm, reduced echogenicity, and a homogeneous structure (Figure 4A). On the right side, the RMB was visualized over a length of 4.25 mm, with a transverse long-axis diameter of 0.9 mm (Figure 4B). The branch extending to the first and SL muscles appeared normal in size and showed no differences between sides (left transverse long-axis diameter = 0.9 mm, right = 0.9 mm) (Figures 4C, 4D). The thickness of the left APB increased to 4.6 mm (right = 5.3 mm), while the thickness of the SL remained consistent between sides (left = 5.64 mm, right = 5.68 mm). The patient remains under observation.

Case 2: Compressive neuropathy of the recurrent motor branch neuropathy of the median nerve following long-distance cycling

A 42-year-old male, measuring 186 cm in height and weighing 91 kg, employed as a lawyer, presented with complaints of numbness in the first to third fingers of his left hand. This symptom emerged after he began regular long-distance cycling, ranging from 10 to 50 km, as a measure to prevent weight gain. He reported no issues with his right hand. His medical history included problems with the left knee joint and presyncopal or syncopal episodes triggered by the sight of blood. He did not experience any pain in the neck or arm.

Neurological examination revealed hypoesthesia in the affected fingers and the radial side of the left ring finger. Despite this, all intrinsic hand muscles, served by both median and ulnar nerves, retained their strength, including the APB. Contrastingly, the right side showed a significant decrease in APB strength (rated 0/5 on the MRC scale), though hand sensitivity remained unaffected, and there was no sign of muscle atrophy or weakness in the muscles innervated by the ulnar nerve.

Neurophysiological testing showed a marked reduction in CMAP amplitude from the APB upon both distal and proximal stimulation on the right side (0.3/0.2 mV), along with slightly delayed distal motor latency (4.4 ms). The left side displayed CMAP amplitudes of 12.8/13.0 mV with a distal motor latency of 3.7 ms. The median antidromic SNAP amplitudes were 77 µV on the right and 54 µV on the left, with conduction velocities of 55 m/s and 54 m/s, respectively. Ulnar SNAP amplitudes were 51.0 µV on the right and 58 µV on the left, with conduction velocities of 64 m/s and 62 m/s.

The findings led to a diagnosis of a compression lesion of the RMB on the right and median nerve compression at the carpal tunnel level on the left due to prolonged cycling. Despite advice to stop cycling, the patient continued his activity. A month later, he experienced numbness in his right hand’s first three fingers but declined further assessment and treatment due to an intolerance to electric current, manifesting as episodes of presyncope.

Case 3: Idiopathic recurrent motor branch neuropathy of the median nerve

A 59-year-old right-handed male reported intermittent numbness in his right little finger, typically after waking up. The numbness was attributed to his habit of sleeping with his arm bent at the elbow. Neurological examination identified hypotrophy in the left thenar and weakness in the first finger’s abduction on the left side, although the patient had not raised any concerns about these symptoms. There was no sensory loss detected, and Tinel, Durkan, Phalen, and reverse Phalen tests returned negative results.

Bilateral ulnar motor and sensory NCSs showed no abnormalities. However, the left APB muscle exhibited slightly prolonged distal motor latency (4.0 ms) compared to the normal side (3.1 ms), with a reduced CMAP amplitude of 4.1 mV against 9.7 mV on the unaffected side. Conversely, conduction studies to the SL muscle were within normal limits, with distal motor latency at 3.3 ms (unaffected side 3.2 ms) and amplitude at 4.7 mV (unaffected side 4.4 mV). The results from all other NCSs were also normal. The findings led to a diagnosis of a mild, isolated lesion of the RMB of the left median nerve, though the cause remained unclear.

Case 4: Compressive neuropathy of the terminal portion of the deep motor branch of the ulnar nerve due to shovel handle impact

A 36-year-old right-handed male exhibited a depression in his right hand’s first dorsal webspace and reported a decline in grip and pinch strength that had appeared three months prior without an apparent cause. He experienced no paresthesia or pain and had no history of hand trauma. Physical examination revealed significant weakness and atrophy of the right FDI muscle but no hypothenar atrophy. Strength in the ADM was normal. Froment’s sign was positive, but Tinel’s, Wartenberg’s, and Egawa’s signs were negative. Pain and tactile sensitivity were normal. An MRI of the cervical spine showed no signs of myelopathy or root compression. NCSs confirmed severe neuropathy of the DBUN, with notably reduced ulnar CMAP amplitude (0.9 mV) and prolonged distal latency (5.2 ms) when recorded over the FDI, whereas ulnar recordings over the right ADM and sensory tests were normal. Motor and sensory NCSs for other nerves were also normal.

During a two-week observation period, the patient was consistently interviewed to uncover the cause of the nerve injury. By the end of this period, he remembered an incident before the onset of atrophy in his first dorsal webspace. While at his country house, the shovel he was using broke. Nonetheless, he continued to dig, holding the shovel’s bottom with his left hand and pressing his right palm against the shovel’s jagged broken end. This led to a blister in the center of his palm, followed by hypotrophy in the first dorsal webspace. This suggests that the neuropathy of the DBUN at the mid-palm level resulted from prolonged and intense pressure from the broken shovel handle on the nerve.

Case 5: Traumatic neuropathy of the median and the deep motor branch of the ulnar nerve caused by a knife injury

**Figure 5:**
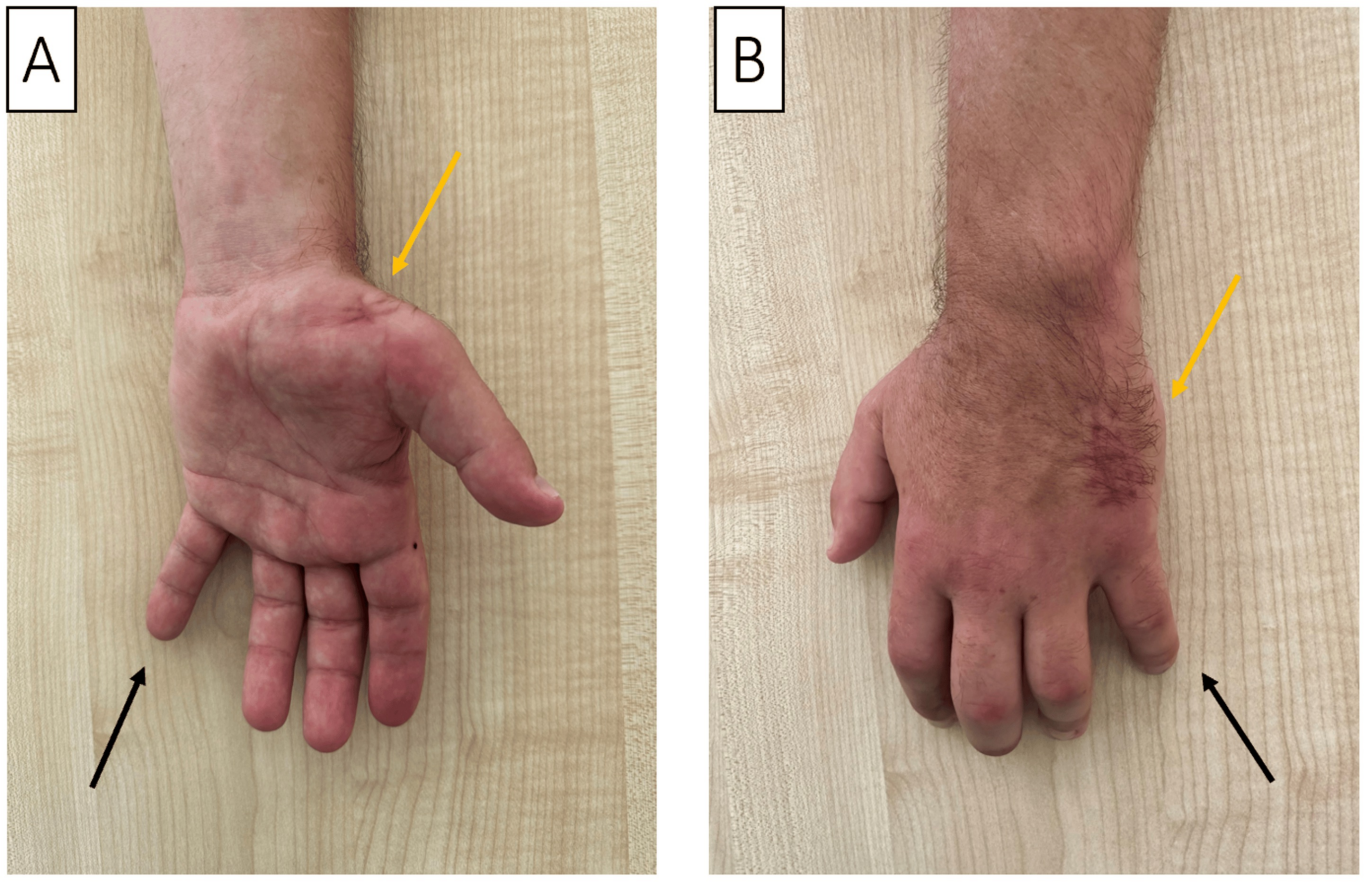
Picture of the patient’s right hand, showing the entry (A, orange arrow) and exit (B, orange arrow) of the blade of a knife. The patient also exhibits atrophy of the thenar eminence and abduction of the fifth digit (Wartenberg sign, black arrow).

A 34-year-old male presented with a complete loss of movement in his left hand and sensory disturbances in the first three digits and the radial side of the fourth digit, following a knife injury incurred while defending against an attack. The blade entered the thenar region and exited on the ulnar side of the hand (Figure 5).

Neurological examination revealed pronounced atrophy of the thenar eminence and the first dorsal webspace, with the fifth digit abnormally abducted, a sign of Wartenberg’s syndrome. The patient could not flex his fingers, nor could he adduct or abduct them, and the thumb was completely immobile. However, abduction strength in the fifth digit was preserved. Sensory examination showed anesthesia in the first and second digits and hypesthesia in the third digit and the radial side of the fourth digit, aligning with the median nerve’s distribution. Tinel’s sign in the middle of the palm elicited a sharp, electrifying pain with tingling that radiated to the first three digits. USI demonstrated evidence of a complete lesion of the first palmar division of the median nerve. No neuroma was identified, and the ultimate cause of the diastasis was impossible to determine.

NCSs highlighted a severe lesion of the left median nerve at the hand, with an absence of CMAP from the left APB and SNAP from the first and second fingers. Partial preservation of CMAP from the SL and SNAP from the third and fourth fingers was noted (Table 2). A severe lesion of the left deep motor DBUN at the palm was also evident, primarily affecting fibers to the FDI and interossei muscles. CMAP from the ADM and SNAP from the fourth and fifth fingers were partially preserved. The injury involved motor fibers of both the median and ulnar nerves, including the first palmar division of the median nerve. Intraoperative findings included fibrotic changes throughout the hand. Procedures performed included tenolysis of the deep flexor tendons of the second and third fingers and suturing of the first palmar division of the median nerve.

**Table 2:** Motor and sensory nerve conduction studies for the fifth patient. CMAP: compound muscle action potential; SNAP: sensory nerve action potential; NR: no response; DML: distal motor latency; CV: conduction velocity; APB: abductor pollicis brevis; SL: second lumbrical; ADM: abductor digiti minimi; IO: interossei; FDI: first dorsal interosseous

Wrist stimulation	Amplitude of CMAP or SNAP, mV or µV		DML, ms or sensory CV, m/s	
Motor studies	Left	Right	Left	Right
Median (APB)	NR	14.6	NR	3.5
Median (SL)	0.4	3.1	3.7	3.2
Ulnar (ADM)	7.1	20.4	2.2	2.3
Ulnar (IO)	0.1	4.2	2.9	2.9
Ulnar (FDI)	0.5	22.4	4.5	2.8
Sensory studies	Left	Right	Left	Right
Median (digit I)	NR	23	NR	50
Median (digit II)	NR	33	NR	56
Median (digit III)	17	28	54	54
Median (digit IV)	9	18	48	54
Ulnar (digit IV)	10	12	54	57
Ulnar (digit V)	15	31	58	58

Case 6: Traumatic neuropathy of the deep motor branch of the ulnar nerve caused by an incident involving a screw and a cordless screwdriver

**Figure 6:**
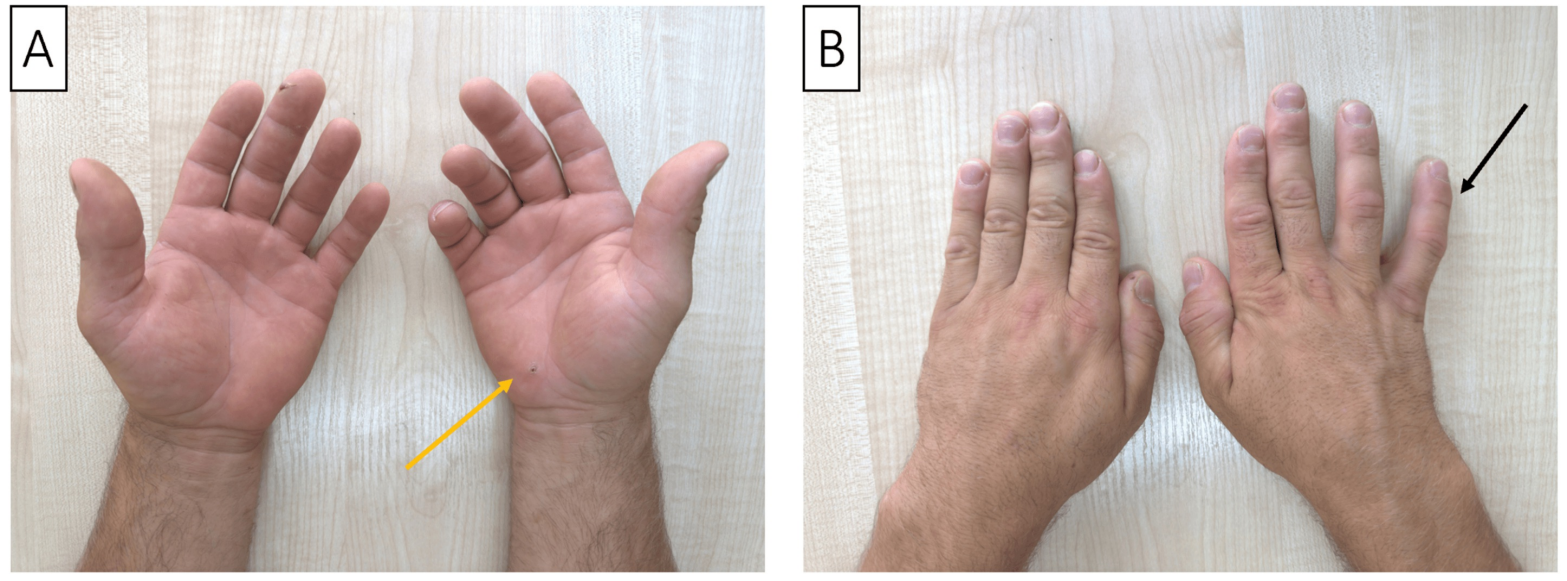
(A) The site of the injury to the ulnar artery and the deep branch of the ulnar nerve (orange arrow). (B) Inability to adduct the fingers of the right hand, in addition to passive deviation of the fifth digit (Warternberg sign, black arrow).

A 34-year-old right-handed male, working as a roofer, experienced a series of symptoms, including clumsiness, slowed finger movements, a progressive decrease in pinch and grasp strength, diminished grip in his right hand, and difficulty picking up screws at first attempt. These issues arose three weeks after an incident while working with a wooden beam and a cordless screwdriver; attempting to drive a screw, it slipped and penetrated the palm of his hand (Figure 6A), leading to significant bleeding that was managed with a tight bandage. On further examination, it was discovered that he had been experiencing numbness in the first three fingers of both hands, predominantly on the right, for one to two years, especially noticeable in the mornings. A physical examination showed a scarred wound on the palm with localized tenderness but no sensory disturbances. The thenar muscles remained strong (5/5 on the MRC scale), whereas the strength in the right hypothenar was slightly reduced (4/5 on the MRC scale). The dorsal and palmar interossei muscles were notably weak (0-1/5 on the MRC scale), preventing finger adduction (Figure 6B). Positive Wartenberg, Froment, and Egava signs were observed, indicating a significant impact on hand function due to the injury.

Right ulnar NCSs showed a decreased CMAP amplitude (8.5 mV compared to 16.6 mV on the unaffected side) when recording from the ADM, and a severely reduced CMAP amplitude from the FDI (0.5 mV compared to 13.9 mV) and the interossei muscles (0.1 mV compared to 4.1 mV), with latencies of 2.8 ms (3.1 ms), 4.3 ms (4.0 ms), and 5.2 ms (3.2 ms), respectively. The SNAPs had normal amplitudes and latencies. Median NCSs indicated prolonged distal CMAP latency (5 ms on the right and 4.7 ms on the left) and decreased sensory conduction velocity (37 m/s on the right and 44.0 m/s on the left). Median distal CMAP latency (recording from the APB and SL) did not differ significantly.

**Figure 7:**
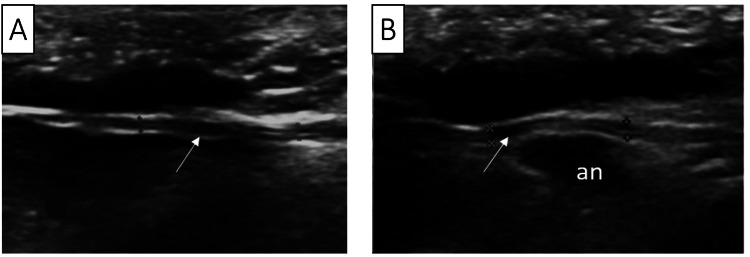
Ultrasound imaging of the deep branch of the ulnar nerve on the unaffected (A) and affected (B) hands. In image A, the nerve path is straight, with no additional formations in the surrounding soft tissues. In image B, the nerve path is irregular, displaced by a round anechoic formation, a pseudoaneurysm.

USI at the forearm level and in Guyon’s canal showed the nerve to be unchanged. The deep branch in the hand was intact but exhibited reduced echogenicity, measuring 1.2 mm in thickness (compared to 0.8 mm on the left), with a heterogeneous structure (Figure 7).

The course of the deep branch was slightly displaced by an additional pulsating formation, a false aneurysm (Video 1). The superficial branch was continuous over its visible extent. The integrity of the ulnar artery was disrupted in the hypothenar area, 1 cm medial to the skin scar, leading to an arteriovenous fistula measuring 7 × 8 mm with circular blood flow (false aneurysm), and a maximum blood flow speed of 3.4 m/s was observed, indicating signs of shunting blood flow in the ulnar artery in the forearm, with “arterialization” of venous blood flow in the forearm’s ulnar veins.

**Video 1:** Ultrasound imaging of the deep branch of the ulnar nerve, showing a pulsating hypoechoic formation disrupting the path of the deep branch of the ulnar nerve, likely causing its compression.

The clinical and electrophysiologic abnormalities, along with USI findings, suggested a lesion affecting the DBUN proximal to the branch supplying the hypothenar muscles, coupled with bilateral, relatively mild CTS. Decompression surgery for the right ulnar and median nerves was performed, revealing the ulnar nerve encased in adhesions. Early follow-up (by three months) confirmed improvement in his ulnar and median nerve symptoms and NCSs. During this time, he underwent two courses of repetitive peripheral magnetic stimulation targeting the hand and the area somewhat proximal to the wrist. The patient remains under observation.

## Discussion

The intrinsic muscles of the hand are crucial for daily activities, particularly those requiring power grasping, pinching, and gripping. The cases explored here highlight various etiological factors and mechanisms of injury to the hand’s motor fibers. All patients with isolated RMB or DBUN damage in this study reported no pain or sensory issues in the median or ulnar nerve territories, despite observable atrophy in the APB or FDI muscles and complaints of hand weakness and clumsiness. However, pain can be observed in the thenar eminence [4].

Diagnosing neuropathies of the median or ulnar nerves beyond the wrist is complex. The hand’s intricate innervation, involving both superficial and deep branches, complicates both clinical assessments and electrodiagnostic examinations. Moreover, the similarity of clinical and electrophysiologic patterns between these neuropathies and other conditions such as CTS, ulnar neuropathy at the elbow, radiculopathy, peripheral neuropathy, and ALS highlights the critical need for precise evaluation and diagnosis [2,7]. A thorough diagnostic approach, integrating clinical and neurophysiological data, with detailed patient history, symptomatology, pathology type, and prognosis, is imperative based on an understanding of the complex anatomy of the hand [6,8].

The median nerve travels through the carpal tunnel before branching into the muscular thenar branch, known as the RMB. Another branch extends into the palm to innervate the first and SL muscles. Its anatomy is unique due to the short distance between where it branches from the median nerve and the motor points of the thenar muscles. Furthermore, the RMB may display variations in its origin and arrangement, sometimes arising as single or multiple branches from the median nerve. Ultrasound studies of the RMB in healthy individuals have recorded a mean transverse diameter of about 0.7 mm ± 0.1, ranging from 0.6 to 1.0 mm, significantly smaller than the 1.4 to 1.7 mm diameters typically noted in anatomical studies [9,10]. The RMB, while small, plays a crucial role in hand function. It innervates key thenar eminence muscles, including the OP, APB, and the superficial part of the FPB, participating in important hand movements such as thumb opposition and palmar abduction of the thumb.

Before the ulnar nerve enters Guyon’s canal, sensory dorsal and palmar cutaneous branches extend from the ulnar nerve. Within Guyon’s canal, there are three anatomical zones: Zone I, proximal, contains both motor and sensory fibers before they bifurcate; Zone II houses the deeper motor fibers; and Zone III, more distal, consists predominantly of sensory fibers [11]. Approximately 1 cm below the pisiform, the ulnar nerve provides a superficial sensory branch that innervates the fingers and motor branches to the palmaris brevis muscle, thereafter forming the DBUN. Further along, the DBUN sends motor branches to the hypothenar muscles, including the abductor, flexor, and opponens digiti minimi. After navigating through the piso-hamate hiatus, it traverses transversely across the palm’s eep aspect at the carpometacarpal joints level. By the third metacarpal, it courses through the adductor pollicis before terminating in branches to the radial interossei. Ultrasound data indicate that the transverse long-axis diameter of the DBUN in healthy volunteers ranges from 0.9 to 2.0 mm, aligning with anatomical studies [12]. It innervates all interossei muscles, the third and fourth lumbricals, the adductor pollicis, and the deep head of the FPB, playing a vital role in finger coordination, grip, and pinch function [1,5,6].

Wu et al. proposed a classification of ulnar neuropathies at the wrist into five types, based on clinical findings, electrophysiological studies, and clinicoanatomic correlations [13]. Type I is a mixed motor and sensory neuropathy, occurring just outside or within the proximal end of Guyon’s canal. Type II is a pure sensory neuropathy, where the lesion affects the superficial branch of the ulnar nerve at the wrist but is distal to the branch to the palmaris brevis. Type III is a pure motor neuropathy due to a lesion of the deep branch of the ulnar nerve just distal to the superficial branch but proximal to the branch to the hypothenars. Type IV is a pure motor ulnar neuropathy with sparing of the hypothenars; this lesion occurs on the deep branch of the ulnar nerve distal to the origin of the superficial branch and distal to the branch going to the hypothenars. Type V is a distal motor neuropathy in which the lesion is just proximal to the branches going to the FDI and adductor pollicis muscles.

Thorough history-taking is essential for pinpointing potential causes of injury and determining the injury’s extent. The distinctiveness of Cases 1 and 4 is not just in the unusual locations of the injuries but also in the patients’ initial failure to connect the significant pain and traumatic impact on their hand tissues to the actual cause. The underlying causes only became evident after meticulously gathering their histories, which included details about their occupations, leisure activities, and actions before the symptoms started. It often required multiple reminders that the injury had to have a direct impact on the nerve at the specific site for the patients to remember the events leading to their conditions. The lack of pain and sensory issues leads patients to remain oblivious to the ongoing nerve compression until the damage becomes severe. Without pinpointing the root cause of nerve damage, the full scope of the condition remains ambiguous.

In some instances, the cause of nerve damage remains unidentified or the issue is discovered incidentally during neurological evaluations. Notably, in two cases (Cases 2 and 3), injuries were found by chance during exams conducted for unrelated concerns. In Case 2, involving a cyclist, a significant RMB injury was detected only during an assessment for sensory disturbances in the opposite hand, later confirmed by NCSs. Case 3 presented a patient where the incidental finding of weakness in palmar abduction of the first finger and thenar hypotrophy lacked an identifiable cause. These symptoms were moderate and did not alarm the patient due to the absence of sensory disturbances, suggesting that such neuropathies might occur more frequently than diagnosed. Chiodo and Chadd (2007) observed that 7 out of 35 hands examined for unrelated symptoms showed ulnar nerve abnormalities upon electrodiagnostic testing [2]. A review of medical literature indicates that the causative factors for injuries to the hand’s motor fibers often remain elusive. Murata et al. reported that 45% of ulnar tunnel syndrome cases in their study were idiopathic, lacking a clear cause for nerve compression [1]. Similarly, Chiodo and Chadd could not ascertain the cause in 12 out of 35 cases of ulnar neuropathy at or distal to the wrist, suggesting cumulative stress rather than trauma as a potential factor [2].

Isolated RMB injuries within the hand are either rare or underdiagnosed, partly due to the lack of imaging techniques capable of accurately visualizing this nerve. Literature documents isolated cases describing RMB injuries from various causes, including cutting injuries from sharp objects, ganglion cysts, entrapment neuropathy from scar tissue, injuries from long-distance cycling, schwannomas, anomalous thenar muscles and blood vessels, and even injuries from intensive hand massages [3,14-18]. Surgical findings have indicated that RMB compression might result from its path through ligaments or an excessive angle at the distal edge of the transverse ligament, causing neuroma formation proximal to the entrapment sites [4,19].

Over 24 months, longitudinal neurological, neurophysiological studies, and USI were conducted on Case 1 who agreed to further investigations. This case stands out due to significant isolated RMB damage, evidenced by thenar muscle atrophy, complete conduction disruption, and an enlargement of the RMB with USI. The NCS data are remarkable. Initially, at months 7 and 11, distal stimulation of the median nerve did not register any CMAP from the APB, showing only a positive curve deviation (Figure 2A). By the 17th month, a distinct bend, corresponding to the initial negative CMAP peak, was noticeable and confirmed by the 21st and 24th months. Reviewing earlier recordings (7 and 11 months) revealed that a very low CMAP was recorded. Over time, these CMAPs’ latency decreased, and amplitude increased, consistent with the clinical picture (Figure 2A). The positive deviation preceding CMAP from the APB during distal and proximal stimulation was likely due to volume conduction from the intact first and SL muscles and forearm muscles (far-field potentials). It had the same latency as CMAP from the SL, and the SL’s negative CMAP peak matched the positive peak deviation timing from the APB. Typically, any positive potential component leads to negative phase cancellation. However, in this case, the CMAP was positioned on the final phase of this positive deviation (distal stimulation) or at some distance from it (proximal stimulation). It is unclear whether volume conduction from the APB influences the low CMAP amplitude recorded from SL in normal conditions (Figure 2B).

In Case 2, both the right and left median nerves were damaged during cycling, likely due to prolonged direct pressure from the handlebars on the median nerve. On the left side, the patient developed CTS with sensory symptoms, which led him to seek medical attention. On the right side, damage to the RMB was detected, characterized by significant weakness in thumb abduction that the patient had not noticed. This muscle weakness, in the absence of atrophy and the lack of CMAP despite preserved sensory fibers, was presumably due to a conduction block in the RMB. Despite being informed about the significant changes observed in the neurophysiological examination, the patient continued cycling, leading to the development of sensory symptoms on the left side, indicative of typical CTS. Whether the RMB injury was an isolated damage or part of the CTS with the anatomical peculiarities of the nerve’s course, remains unclear.

To date, there has been only one reported case of bilateral RMB damage following long-distance cycling [16]. Typically, cycling leads to distal ulnar neuropathies in Guyon’s canal. The bilateral nature of the injuries in that case suggests either an unusual amount of pressure from the handlebars or an atypical anatomy of the patient’s hand and/or the position of the RMB. A case of CTS with sensory disturbance post-cycling was also documented [20]. However, a study by Akuthota et al. found no significant electrophysiologic abnormalities in median motor and sensory functions in a group of 14 cyclists after a six-day, 420-mile bike tour [21].

Electrophysiological and clinical studies have highlighted the potential for CTS to exclusively involve motor fascicles or to represent a condition distinct from classical CTS symptoms. Exclusive motor fiber involvement in CTS is uncommon, seen in only about 1.2% of cases [22]. Anatomical variations in blood vessels and the origin and course of the RMB might predispose the nerve to impingement [10,18]. Yet, the precise prevalence and impact of RMB neuropathy in patients with CTS remain to be fully determined. Riegler et al. proposed the use of high-resolution ultrasound as a future method for detecting such conditions [9]. Additionally, the RMB is particularly vulnerable to iatrogenic injuries during decompression surgeries for CTS, owing to its anatomical variability, especially concerning its origin from the median nerve and its path relative to the transverse carpal ligament. Iatrogenic injuries to the RMB, sometimes referred to as the “million-dollar injury,” can lead to significant legal settlements due to the loss of thenar muscle function [9]. This highlights the critical need for thorough preoperative evaluations of these patients to mitigate inherent risks.

DBUN damage occurs more frequently than RMB damage of the median nerve, though the precise frequency of both injuries is still unknown. A review of medical literature identifies a variety of causes and risk factors for DBUN damage, including local masses (ganglion cysts, schwannomas), trauma from long-term pressure (such as from crutches, vibrating tools, and bicycle handlebars), anatomical abnormalities, carpal and metacarpal fractures or dislocations, arthritis, scarring, flexor tenosynovitis, fibrous bands, complications from open carpal tunnel release surgery, leash of vessels, and complications related to the ulnar artery such as thrombosis or aneurysm [1,2,5-7,23-28].

Thrombosis or aneurysm of the ulnar artery, typically occurring in Zone II, where injury to the ulnar nerve due to direct trauma to the ulnar artery in its confined and vulnerable position has been previously reported as part of the hypothenar hammer syndrome [11]. The mechanism of this injury involves a direct blow causing the artery to hammer against the hook of the hamate, acting as an anvil, leading to artery damage with thrombosis and occlusion, resulting in ischemia and related ulnar nerve symptoms, such as sensory disturbances. In our Case 6, a traumatic and compressive mechanism of DBUN injury was apparent due to direct trauma and pressure from the false aneurysm and surrounding adhesions. The localization of the damage aligns largely with type III of Wu et al.’s classification [13].

Case 4 discussed mid-palmar localization of DBUN damage caused by external compression of the nerve, a rare occurrence. External compression on the palmar surface of the hand causing palsy of the terminal motor branch is notably unique. Common causes of distal ulnar nerve compression usually involve ganglion cysts originating from the pisotriquetral joint. Duggal et al. reported a unique case of a complex ganglion originating from the third carpometacarpal joint causing compression of the DBUN [29]. De Maio et al. reported a case of isolated paralysis of the adductor pollicis due to a fibrous band joining the pisiform and the hook of the hamate bone that compressed the DBUN [23]. Jennings and Jennings reported three cases of damage to the DBUN at the level of the third metacarpal (intraneural ganglion cyst, leash of three vessels just radial to the third metacarpal, series of fibrous bands) [6]. As the source of compression was identified near the level of the adductor hiatus in each of these patients, all had a positive Froment sign, a positive Egawa sign, and a negative Wartenberg sign with concomitant wasting of the FDI muscles.

A similar clinical picture was detected in our patient. The localization of the damage corresponds to type V of Wu et al.’s classification [13]. Generally, ganglion cysts at the mid-palmar region causing ulnar nerve compression are very rare [30]. Yanagisawa et al. (2021) reported a rare case where a 39-year-old woman presented with a ganglion cyst in the carpal tunnel, simultaneously compressing the right median nerve and the DBUN [17]. Furthermore, a rare case reported simultaneous compression of the right median nerve and DBUN by a ganglion cyst in the carpal tunnel. Our Case 5 showcased combined traumatic damage to both RMB and DBUN, alongside a complete transection of the first sensory palmar branch of the median nerve due to a knife wound.

The treatment outcomes for isolated motor nerve injuries of the hand varied based on the cause and severity of the neuropathy, as well as the intervention applied (Table 3). In cases such as the traumatic neuropathy of the RMB of the median nerve (Case 1) and the combined median and DBUN neuropathy due to a knife injury (Case 5), targeted interventions including medications, physical therapy, and surgical repair led to significant functional recovery, although some residual deficits remained. Conservative management in idiopathic and compressive neuropathies (Cases 2, 3, and 4) resulted in the stabilization of symptoms, with patients adapting to mild functional limitations. In the traumatic DBUN neuropathy caused by a screw injury (Case 6), surgical decompression and repetitive peripheral magnetic stimulation markedly improved nerve function and reduced symptoms. These findings underscore the importance of early and accurate diagnosis, as well as a tailored treatment approach, in enhancing patient outcomes in isolated hand motor nerve injuries.

**Table 3:** Summary of patient demographics, causes, NCS/USI results, treatments, and outcomes. NCS: nerve conduction study; USI: ultrasound imaging; PT: physical therapy; RMB: recurrent motor branch; DBUN: deep motor branch of the ulnar nerve; APB: abductor pollicis brevis; FDI: first dorsal interosseous

Case	Age	Sex	Profession	Cause	NCS/USI Results	Treatment	Outcome
1	42	M	Automobile mechanic	Blow to the thenar from a knife blade	Severe axonal damage to the median nerve, significant atrophy on USI	Medications, PT, magnetic stimulation	Partial recovery of thumb function
2	42	M	Lawyer	Long-distance cycling	Compression lesion of RMB on the right, median nerve compression on the left	Advised to stop cycling	Continued cycling, no further treatment
3	59	M	Unknown	Idiopathic	Mild lesion of RMB of the left median nerve	Conservative management	No significant improvement or worsening
4	36	M	Unknown	Impact from a broken shovel handle	Severe neuropathy of DBUN, atrophy of FDI on USI	Conservative management	Adapted to mild functional limitations
5	34	M	Unknown	Knife injury	Severe lesion of median and DBUN, fibrotic changes, atrophy on USI	Surgery, PT	Partial recovery of hand function
6	34	M	Roofer	Screwdriver incident	Lesion affecting DBUN, mild carpal tunnel syndrome	Surgery, magnetic stimulation	Improved ulnar and median nerve symptoms

Given the dearth of information in the literature regarding the diagnosis and approach to the evaluation of isolated motor nerve injuries, this study provides valuable insights. Our comprehensive approach to diagnosing and evaluating isolated motor nerve injuries in the hand, integrating clinical evaluation, standardized questionnaires for detailed medical history, electrophysiological testing, and high-resolution USI highlights a conceptual framework that can be used to diagnose complex neuropathies. We readily concede that this study has several limitations. The study’s retrospective nature and the small sample size limit the generalizability of the findings. Additionally, as the cases were selected retrospectively from medical records, there is a possibility of selection bias, and the retrospective design precludes drawing causal conclusions. Future studies with larger, prospective cohorts are needed to validate these findings and explore the generalizability of the results presented.

## Conclusions

In our study, we highlighted diverse cases of motor hand neuropathies. The complexity of the diagnosis is emphasized, particularly due to the absence of pain and sensory disturbance, which means patients often remain unaware of ongoing nerve compression until significant damage occurs. The comprehensive medical history stands out as a crucial element, providing key insights into the mechanism behind the injury’s development. Furthermore, electrophysiological tests, combined with imaging modalities, can provide a comprehensive understanding of these neuropathies. Early therapeutic interventions, from removing causative factors to surgical decompression, are vital for preventing further damage and restoring function.
